# Ascending Trouble: Guillain-Barré-Like Syndrome Due to West Nile Virus

**DOI:** 10.7759/cureus.85240

**Published:** 2025-06-02

**Authors:** Patricia E Simmer, Victoria Powell, Virginia Hoch, Christian Noblett, Patrick Eckert, Peter Abdelmaseeh

**Affiliations:** 1 Emergency Medicine, Internal Medicine, Christiana Care Health System, Newark, USA; 2 Internal Medicine-Pediatrics, Christiana Care Health System, Newark, USA; 3 Internal Medicine, Christiana Care Health System, Newark, USA; 4 Critical Care Medicine, Christiana Care Health System, Newark, USA

**Keywords:** clinical case report, gbs mimics, guillain-barré-like syndrome, guillain-barre syndrome (gbs), west nile virus infection

## Abstract

Guillain-Barré syndrome (GBS) and GBS-like syndromes secondary to West Nile virus (WNV) are rare and poorly characterized. GBS is an immune-mediated disease that affects peripheral nerves, classically in a symmetrical and ascending pattern. It is generally associated with infectious microbes, but not classically with WNV. WNV, although a relatively new pathogen in the United States, has become a leading mosquito-borne viral infection in the country. We present a case of a patient who came to the hospital in Delaware, USA, with fever and confusion that rapidly progressed to worsening encephalopathy and paralysis requiring intubation and vasopressor support. This case emphasizes the need to include GBS or GBS-like syndromes on the differential in patients with encephalopathy and neurological changes, particularly in the setting of an increasing incidence of WNV infection in the United States.

## Introduction

Guillain-Barré Syndrome (GBS) and GBS-like syndromes secondary to West Nile Virus (WNV) are rare and poorly characterized. GBS itself is rare, with an estimated 100,000 new cases per year [1]. GBS, an immune-mediated disease that affects peripheral nerves, classically in a symmetrical and ascending pattern [2], is generally associated with infectious microbes, including Campylobacter jejuni, Haemophilus influenzae, Mycoplasma pneumoniae, cytomegalovirus (CMV), Epstein-Barr virus (EBV), hepatitis E virus, influenza, and Zika virus [3]. WNV is a leading mosquito-borne viral infection in the United States [4]. There are a variety of neurologic syndromes that can be seen with WNV, and although not classically associated with GBS or GBS-like syndromes, there is a small incidence of WNV-associated GBS estimated at 0.6/100,000 cases annually [5].

We present a case of a patient with an unusual initial presentation with complications, including rapid neurological deterioration requiring intubation and vasopressor support, who was subsequently diagnosed and treated for a WNV-associated GBS-like syndrome.

## Case presentation

**Figure 1:**
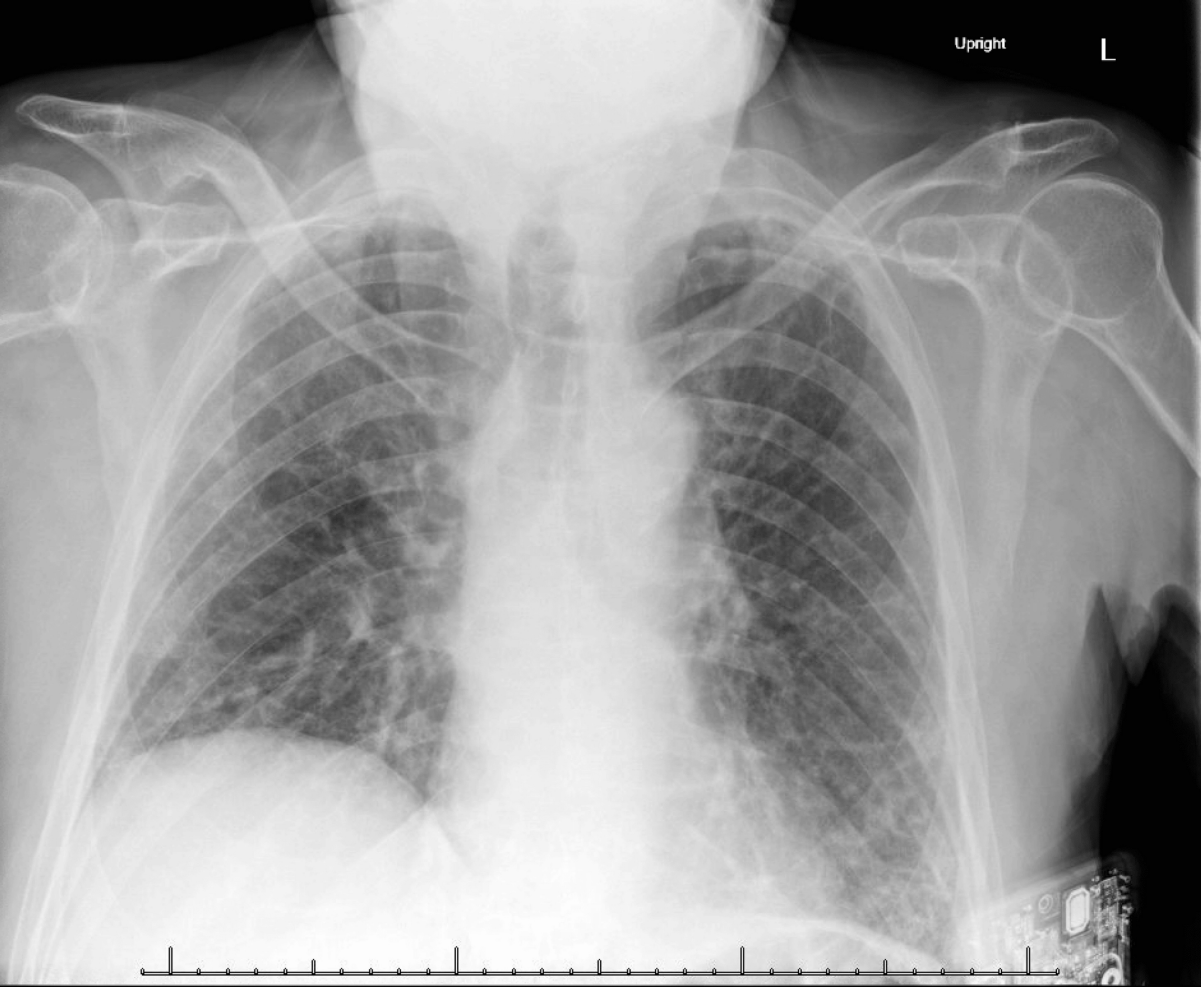
Chest X-ray demonstrating bibasilar reticular interstitial opacities, consistent with known interstitial lung disease.

A 73-year-old man with a history of interstitial lung disease (ILD) on chronic prednisone (10 mg daily), long COVID-19, and pulmonary embolism presented to the hospital in August with several days of fatigue, cough, drowsiness, and difficulty walking. On presentation, he was found to be hypotensive (blood pressure 90/62 mmHg) and intermittently confused. He was noted to have a Glasgow Coma Score of 15, although there were instances where he required re-orientation to place and time. He had an otherwise normal neurologic exam. Of note, he had no recent travel history, but one week prior, he had experienced a fever and diarrhea. Initial laboratory and imaging workup was notable only for hyponatremia (127 mmol/L) and bibasilar opacities on chest X-ray, consistent with ILD (Figure 1).

He was admitted for workup of encephalopathy and started on antibiotics for suspected pneumonia. Subsequent neurologic exams were notable for increasing muscular fasciculations, as well as ongoing confusion. Mental status was described as waxing and waning, with brief periods during which he became less talkative and occasionally exhibited a stare. As part of the workup, an electroencephalogram (EEG) was obtained. The official read noted slowing in the left temporal region and mild generalized background slowing. EEG images were not available for inclusion in this report. Cerebrospinal fluid (CSF) was obtained via lumbar puncture. CSF analysis results are as shown in Table 1.

**Table 1:** Cerebrospinal fluid (CSF) analysis results.

Laboratory	Laboratory value	Reference range (units)
White blood cell (WBC) count	76/CUMM	0-5/CUMM (cells per cubic millimeter)
Neutrophils (%)	29%	
Lymphocytes (%)	52%	
Macrophage (%)	19%	
Protein	84 mg/dL	15-45 mg/dL
Glucose	70 mg/dL	40-70 mg/dL

**Figure 2:**
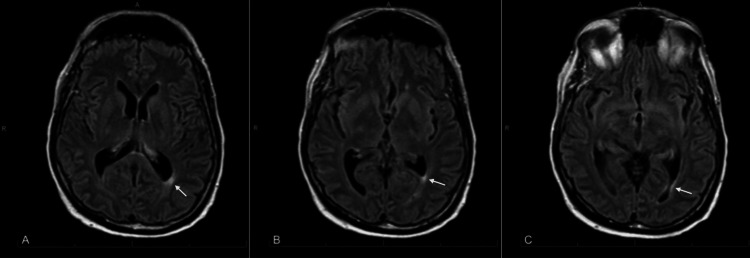
Magnetic resonance imaging (MRI) brain. T2-weighted images with fluid-attenuated inversion recovery (T2/FLAIR) axial view demonstrating minimal nonspecific periventricular white matter gliosis, most notable on the left. Images labeled A, B, and C are sequential coronal cuts, with A being the most cranial and B and C progressing caudally.

The patient underwent brain magnetic resonance imaging (MRI) with contrast, which showed no pathologic enhancement. Minimal nonspecific periventricular white matter gliosis was present, particularly abutting the left atrial trigone (Figure 2).

After three days in the hospital with no improvement, antimicrobial coverage was broadened to cover tick-borne illness and Herpes Simplex Virus (HSV). His steroid dose was also increased from his home dose of prednisone 10 mg daily to methylprednisolone 20 mg daily, but his confusion, tremulousness, and fasciculations continued to worsen. One week into admission, his neurological status deteriorated further, with the development of quadriparesis (strength reported as 1-2/5 in all extremities) as well as areflexia. He suffered an aspiration event with loss of cough reflex and swallowing ability. He was intubated and transferred to the intensive care unit (ICU) for respiratory and vasopressor support in the setting of acute respiratory failure and shock of unclear etiology. At this point differential diagnosis for neurologic symptoms was broad. An extensive infectious and autoimmune workup was performed, including CSF meningitis/encephalitis screening by polymerase chain reaction (PCR), serum cryptococcal antigen, Lyme titer, antinuclear antibodies (ANA), and a vasculitis panel. All of these tests were ultimately negative. Alternative diagnoses such as botulism, St. Louis encephalitis, dengue fever, or non-infectious causes such as spinal cord infarct or central pontine myelinolysis were not considered. Documentation upon transfer to the ICU noted absent sensation and 0/5 strength in the bilateral lower extremities, with weak but present hand grip in the bilateral upper extremities. He was able to move his head and neck with a full range of motion. Given the ascending pattern, there was clinical concern that his presentation was consistent with GBS or GBS-like syndrome. He was started on intravenous immunoglobulin (IVIG) therapy. Electromyography (EMG) was considered, but this was not pursued. Neurology documentation indicated that EMG results would not alter the treatment course or affect the outcome. WNV IgM titers later returned positive in both serum and CSF. The patient completed a five-day course of IVIG and exhibited slow improvement in mental status and strength. He ultimately required tracheostomy and gastrostomy tube placement and was discharged after approximately five weeks in the hospital to a skilled nursing facility for further rehabilitation. Upon discharge, his neurologic exam noted generalized weakness with the ability to move all four limbs against gravity.

## Discussion

WNV is an RNA flavivirus, naturally occurring in birds and transmitted to humans in a mosquito-borne fashion. It has only been endemic in the United States since around the turn of the century. The first cases were reported in 1999, and since then, there have been an estimated 7 million human infections [6]. The majority of people infected are asymptomatic or merely develop a mild febrile illness. However, a small percentage (<1%) develop neuroinvasive complications. In 2024, there have been over 800 reported cases of neuroinvasive human disease in the United States [7].

Neuroinvasive WNV is typically seen in older or immunosuppressed patients [8], as in our case of a 73-year-old man on chronic prednisone therapy for interstitial lung disease. Neuroinvasive WNV typically involves the central nervous system, specifically anterior horn cells in the spinal cord and, less frequently, motor neurons in the brainstem [9]. Diagnosis is usually made with specific IgM antibodies detected in blood or CSF. CSF also typically shows increased leukocytes with at least 50% neutrophils, increased protein, and normal glucose [10]. Our patient did have elevated protein and leukocytes in his CSF, but did not have a neutrophil predominance.

Neuroinvasive WNV also may produce characteristic imaging findings on MRI, generally areas of increased signal intensity on T2/FLAIR sequences [8]. Our patient did not exhibit any abnormalities on MRI of the brain, although he never underwent spinal cord MRI, which may also show abnormalities and could distinguish neuroinvasive WNV involving the anterior horn from a syndrome of peripheral neuron involvement, such as in GBS.

Symptoms of neuroinvasive WNV include encephalitis or acute flaccid paralysis [11]. This paralysis is most frequently asymmetric and is secondary to a poliomyelitis of anterior horn cells, resulting in widespread asymmetric muscle denervation without demyelination or myopathy. Electrophysiologic studies, such as EMG or nerve conduction studies (NCS) in these cases, help distinguish anterior horn axonal processes from peripherally demyelinating syndromes such as GBS or other peripheral nerve disorders [9]. EMG is more sensitive 7-14 days after symptom onset; however, despite the patient’s prolonged hospital stay, EMG was not performed during this period. Table 2 provides a review of the pathophysiology, clinical presentations, and treatment of the similar but distinct disorders neuroinvasive WNV, GBS, Lyme neuroborreliosis, and neuroinvasive poliovirus [5,8-10,12-16].

**Table 2:** Comparison of various conditions associated with viral illness that may present with or result in flaccid paralysis: neuroinvasive West Nile virus (WNV), Guillain-Barré syndrome (GBS), Lyme neuroborreliosis (also referred to as Bannwarth syndrome), and neuroinvasive poliovirus infection. [5,8-10,12-16]. Please note that this is not a comprehensive list of conditions that may result in flaccid paralysis. We do not include details of various toxins or central (brain or spinal cord) conditions that may present with similar symptoms. WNV, West Nile Virus; GBS, Guillain-Barré Syndrome; CSF, cerebrospinal fluid; WBC, white blood cell; MRI, magnetic resonance imaging; T2/FLAIR, T2-weighted and fluid attenuated inversion recovery; PCR, polymerase chain reaction; RNA, ribonucleic acid; IVIG, intravenous immunoglobulin

	Neuroinvasive WNV	GBS	Lyme neuroborreliosis	Neuroinvasive poliovirus
Pathophysiology	Direct viral infection of motor neurons in the anterior horns of the spinal cord	Immune destruction of peripheral nerves, triggered by a response to antigens from antecedent infection	Spirochete invasion in the leptomeninges, nerve roots, and dorsal root ganglia, with subsequent inflammation	Viral invasion and destruction of alpha motor neurons in the anterior horns of the spinal cord, cranial nerve nuclei of the medulla oblongata, pons, and midbrain
Symptoms	Acute asymmetric weakness, usually without sensory findings. Sometimes long-term sequelae persist weeks after infection, including muscle fasciculations or atrophy, tremors.	Symmetrical ascending flaccid paralysis. Sometimes associated with hypo/areflexia, non-length dependent neuropathies, dysphagia, and dysautonomia.	Painful radiculitis is most common, along with cranial palsy (mostly facial palsy). Can be accompanied by peripheral nerve paresis, or myelitis with spastic ataxic gait and bladder dysfunction.	Asymmetric weakness or flaccid paralysis classically progresses from proximal to distal.
Studies	CSF: pleocytosis (WBC count average >200) with neutrophil predominance	CSF: classical finding of albuminocytologic dissociation (normal WBC count and elevated protein level)	CSF: elevated WBC count (usually lymphocytes, monocytes, or plasma cells), elevated protein, Borrelia-specific intrathecal antibodies, or PCR-detectable Borrelia species antigens. Seen in about 50% of cases.	Diagnosis with viral culture or detection of viral RNA in stool or throat irrigation/swab
	MRI: foci of increased signal intensity on T2/FLAIR sequences in the thalamus and brainstem, as well as spinal cord	MRI: enhancement of nerve roots	MRI: nonspecific abnormal findings predominantly within the frontal cortex, white matter, and arcuate fibers.	
		Electromyography (more sensitive 7-14 days after symptoms): absent/prolonged H-reflexes and/or F-wave latencies with sural sparing		
Treatment	Supportive	IVIG, Plasma exchange	Antibiotic therapy	Supportive
Natural history	Neurological symptoms present concurrently with initial infectious symptoms. May have long-term neurologic sequelae.	Monophasic (not relapsing/remitting) course within one to six weeks of antecedent illness	Following erythema migrans after a tick bite, it may last three to five months.	Initial infectious symptoms, followed several days later by meningitis; then apparent recovery, followed by relapse of infectious symptoms and appearance of neurologic symptoms.

GBS or GBS-like syndromes are rare but known complications of WNV that have clinical presentations distinct from neuroinvasive WNV. GBS affects the peripheral nervous system and results in a classical syndrome of ascending paralysis associated with sensory changes, progressing to respiratory failure requiring intubation in 20%-30% of cases [15,17,18]. Our patient exhibited a symmetric ascending paralysis, ultimately resulting in respiratory failure and the need for intubation. There is no documented evidence before transfer to the ICU that our patient experienced any sensory changes, and his CSF studies did not show the classic findings seen in GBS: albuminocytologic dissociation (normal WBC count and elevated protein level). While this patient’s diagnosis was not immediately clear and not consistent with classic GBS, his clinical pattern of ascending and progressive paralysis ultimately prompted the initiation of therapy with IVIG for treatment of a GBS-like syndrome.

GBS is treated with IVIG or plasma exchange, which have similar effectiveness and safety profiles [17,19,20]. Our patient received five days of IVIG therapy initiated after the onset of weakness. Despite therapy, many patients, including ours, suffer a long disease course and recovery time, taking months to years to recover functional status, although there is evidence to suggest better outcomes with earlier initiation of treatment (within two weeks of onset of weakness) [17].

## Conclusions

Historically, WNV has been considered rare, but it is now a common mosquito-transmitted virus in the United States. Although the majority of patients have minimal symptoms or are asymptomatic, there is potential for neuroinvasive disease. We present a case of significant neurologic complications associated with WNV, thought to be in the setting of a GBS-like syndrome. Although it is difficult to say for certain that this was not purely a case of neuroinvasive WNV-associated anterior horn pathology (the treatment for which is supportive), a clinical diagnosis of GBS-like syndrome was made and resulted in prompt treatment with IVIG, at which point the patient started to exhibit clinical improvement. Neuroinvasive WNV associated with GBS has a high risk of progression to life-threatening complications such as respiratory failure requiring intubation. Further, there is evidence to suggest prompt treatment improves functional outcomes and recovery times in patients with GBS. Given the severity of the disease, the potential for increasing incidence, and the importance of timely clinical recognition and treatment initiation, neuroinvasive WNV leading to GBS or GBS-like syndrome should be considered in patients presenting with encephalopathy and neurological changes.
